# Risk factors for postoperative DVT exacerbation or new DVT in patients with spinal cord injury complicated with cervical fracture

**DOI:** 10.3389/fcvm.2024.1458941

**Published:** 2024-09-30

**Authors:** Haiying Wang, Bing Lv, Wei Li, Jingjing Xu, Ce Ma

**Affiliations:** ^1^ Department of Orthopaedic Surgery, Baoding No.1 Central Hospital, Baoding, China; ^2^ Department of Ultrasound Medicine, Baoding No.1 Central Hospital, Baoding, China

**Keywords:** deep venous thrombosis, spinal cord injury, cervical fracture, risk factor, thrombosis exacerbation

## Abstract

**Objective:**

To investigate the dynamic changes of perioperative deep venous thrombosis (DVT) in patients with spinal cord injury complicated with cervical fracture and analyze the risk factors of postoperative DVT exacerbation or new DVT.

**Methods:**

From January 2018 to December 2023, a total of 232 patients with spinal cord injury complicated with cervical fracture in our hospital were retrospectively analyzed. Ultrasonography of both lower limbs was performed before and after surgery. According to whether there was aggravation of DVT or new thrombosis, the group was divided into DVT exacerbation or new DVT group and non-DVT exacerbation group. Clinical data were used to study the dynamic changes of perioperative DVT. Logistic regression analysis and receiver operating characteristic (ROC) curve were used to explore the risk factors.

**Results:**

The DVT was found in 47 patients before surgery, including 26 cases of distal thrombus, 14 cases of mixed thrombus and 7 cases of proximal thrombus. Postoperative DVT increased to 81, including 31 distal thrombus, 35 mixed thrombus and 15 proximal thrombus. A total of 53 patients (22.8%, 53/232) experienced postoperative DVT exacerbation or new DVT. Logistic regression analysis revealed that age, American Spinal Injury Association (ASIA) score, time from injury to surgery, surgery time and blood loss were risk factors for postoperative DVT exacerbation.

**Conclusions:**

In patients with spinal cord injury complicated with cervical fracture, the risk of postoperative DVT exacerbation is very high. Age, ASIA score A-B, time from injury to surgery, surgery time and blood loss were risk factors for postoperative DVT exacerbation or new DVT.

## Introduction

Cervical fracture with acute spinal cord injury (SCI) is common following cervical spine trauma and is associated with a number of complications (1). The prevalence of SCI with cervical fracture is 10%–51% (2–4). Cervical fracture with SCI can result in high mortality, severe disability, central nervous pain, and significant socioeconomic burden (5–7). Patients with acute SCI are at high risk for venous thromboembolism (VTE), which includes DVT and pulmonary embolism (PE). The incidence of VTE in patients with acute SCI is 12%–64%, and the mortality rate is 9.7% (8, 9).

The DVT refers to abnormal coagulation of blood in the deep venous cavity, blocking the venous lumen and causing venous obstruction, which is commonly seen in the lower limbs (10). Although DVT has obvious clinical manifestations such as pain in the early stage, the main complaints of patients with SCI are not obvious due to partial or complete loss of superficial sensation. 50%−80% of patients with DVT had no specific clinical symptoms in the early stage, and over 50% of patients had normal physical examinations, leading to a high rate of missed diagnosis (11). So, there are significant differences in the epidemiology of DVT in patients with SCI reported by different studies.

Many studies have investigated risk factors associated with DVT in patients with cervical SCI (12–16). Previous studies mainly focus on static DVT after SCI. But to our knowledge, after the formation of DVT in patients with SCI, only a few thrombi are confined to the site of occurrence or disappear spontaneously, and most of them extend to the deep venous trunk of the entire limb, which can cause various complications such as post-thrombotic syndrome, edema, compression ulcer and even PE (17–19). There are no reports on the dynamic change of DVT and the risk factors of thrombosis aggravation in patients with cervical SCI.

The purpose of this study was to investigate the dynamic change of perioperative DVT in patients with SCI complicated with cervical fracture and analyze the risk factors of postoperative DVT exacerbation or new DVT.

## Patients and methods

### Study population

This retrospective study analyzed the data of patients with acute SCI complicated with cervical fractures admitted to Baoding No.1 Central Hospital from January 2018 to December 2023. Finally, a total of 232 patients were enrolled, including 175 males and 57 females. The causes of injuries included 114 cases of high fall injuries, 82 cases of traffic accidents, 14 cases of fall at ground level and 22 cases of injury caused by heavy object. The study inclusion criteria were as follows: (1) acute SCI patients with cervical fractures admitted to our hospital within 1 week of injury caused by trauma; (2) complete medical records. The exclusion criteria were as follows: (1) chronic SCI patients(>1 week); (2) combined with multiple organ injury; (3) abnormality of the coagulation function; (4) anticoagulation before injury; (5) previous history of VTE; (6) accompanied by other sites of fractures. This study was approved by the Ethics Committee of our hospital and our study obtained the informed consent of all patients.

### Diagnosis, prevention and grouping of DVT

Ultrasonography was conducted to diagnose DVT. The examination encompassed all veins in the lower extremities, including the external iliac vein, femoral vein, popliteal vein, posterior tibial vein, fibular vein, and intermuscular vein. All examinations were performed by qualified radiologists using the same ultrasound equipment (Philips Medical Healthcare, Armstrong, Netherlands). All patients underwent lower limb venous ultrasonography before and 3–5 days after surgery. The diagnosis of DVT is based on the Guidelines for Diagnosis and Treatment of Deep Venous Thrombosis (Third Edition) proposed by the Chinese Medical Association (20). The DVT was categorized into three distinct types: central DVT (occurring in the popliteal vein or proximal regions), peripheral DVT (localized distally to the popliteal vein), and mixed DVT (involving both central and peripheral thrombosis).

All patients in this study received both mechanical and chemical prophylaxis after admission (21). Low molecular weight heparin (LMWH) (4,100 U, once per day, Changshan Production, Hebei, China) was subcutaneously injected to prevent DVT. Mechanical prophylaxis, such as intermittent pneumatic compression and ankle pump training, was also implemented. Patients with DVT received twice daily treatment with LMWH and immediately stopped mechanical prophylaxis. It was up to the vascular surgeon to decide whether to use an inferior vena cava filter. The anticoagulation strategy remained unchanged after surgery.

Patients were divided into DVT exacerbation or new DVT group and non-DVT exacerbation group based on dynamic change in thrombosis detected by ultrasonography. The DVT exacerbation or new DVT group was defined as a new proximal thrombus, a new mixed thrombus, a new distal thrombus, a single thrombus developing into a double thrombus, and a distal thrombus progressing to a proximal thrombus or mixed thrombus.

### Data collection

Demographic and clinical data were collected, including age, gender, height, weight, body mass index (BMI), comorbidities (including hypertension, diabetes and coronary heart disease), ASIA score, time from injury to surgery, causes (including traffic accident, high fall, injury caused by heavy object, and fall at ground level), surgery time, blood loss, liquid transfusion, blood transfusion, surgical procedures (anterior surgery or posterior surgery), D-dimer, fibrinogen(FIB), prothrombin time(PT), thrombin time(TT), activated partial thromboplastin time(APTT).

### Statistical analysis

Statistical analyses were conducted with SPSS (Version 22.0, IBM SPSS Inc., Chicago, IL, USA). Continuous variables with normal distribution were given as mean ± standard deviation (SD）and compared using independent *t* tests. Non-normally distributed continuous variables were represented as median values (interquartile range, IQR) and examined using the Mann-Whitney *U*-test. Categorical variables were presented as proportions and compared using the chi-squared test. The variables showing *p* < 0.05 were selected into the multivariate logistic analysis which explored risk factors for DVT exacerbation. ROC curve analyses were also used to evaluate the diagnostic value of risk factors. *P*-values <0.05 were considered statistically significant.

## Results

### Perioperative change of DVT before and after surgery

174 patients (75.00%, 174/232) had no change in thrombus before and after surgery (including 5 patients with proximal thrombus, 11 patients with mixed thrombus, 10 patients with distal thrombus and 148 patients without thrombus). Among 37 patients without thrombus before surgery (15.95%, 37/232), there were 6 cases of proximal thrombus, 16 cases of mixed thrombus, and 15 cases of distal thrombus after surgery. Four patients (1.72%, 4/232) with distal thrombus changed to proximal thrombus after surgery, and nine patients (3.88%, 9/232) changed to mixed thrombus. Three patients (1.29%, 3/232) of mixed thrombus became distal thrombus after surgery, and 1 patient had thrombus disappearance after surgery (Table 1).

**Table 1:** Dynamic changes of DVT before and after surgery.

Preoperative thrombosis	Cases(*n*)	Postoperative thrombosis			
		Proximal thrombus	Distal thrombus	Mixed thrombus	No thrombus
Proximal thrombus	7	5	1	1	0
Distal thrombus	26	4	11	9	2
Mixed thrombus	14	0	3	10	1
No thrombus	185	6	16	15	148
Total	232	15	31	35	151

### Univariate analysis of DVT exacerbation or new DVT

As shown in Table 2, the average age in the DVT exacerbation or new DVT group was 57.94 ± 11.38 whereas that in the non-DVT exacerbation group was 44.07 ± 13.10. The patients with DVT exacerbation were older than those without DVT exacerbation (*P* < 0.001). There was a significant difference in ASIA score between the two groups (*P* < 0.001). Time from injury to surgery was significantly different between the two groups: 7.62 ± 2.93 vs. 5.51 ± 2.68, respectively (*P* < 0.001). there were also significant differences in surgery time, blood loss, blood transfusion, and D-dimer between the two groups (all *P* < 0.05). However,there were no significant differences between the two groups in gender, BMI, comorbidities, causes, surgical procedures, FIB, TT, APTT, and PT (all *P* > 0.05).

**Table 2:** Patient characteristics between the two groups.

Variables	DVT exacerbation or new DVT Group (*n* = 53)	Non-DVT exacerbation Group (*n* = 179)	*P* Value
Age(years)	57.94 ± 11.38	44.07 ± 13.10	<0.001
Gender(male/female)	38/15	137/42	0.472
BMI(kg/m^2^)	24.12 ± 1.96	23.80 ± 2.14	0.325
Comorbidities			
Hypertension(%)	12/53 (22.64%)	54/179 (30.17%)	0.286
Diabetes(%)	7/53 (13.21%)	27/179 (15.08%)	0.734
Coronary heart disease(%)	2/53 (3.77%)	11/179 (6.15%)	0.510
Causes			
Traffic accident	22 (41.51%)	60 (33.52%)	0.612
Fall at ground level	4 (7.55%)	10 (5.58%)	
Injury caused by heavy object	5 (9.43%)	17 (9.50%)	
High fall	22 (41.51%)	92 (51.40%)	
ASIA score			
A–B	25 (47.17%)	24 (13.41%)	<0.001
C–D	28 (52.83%)	155 (86.59)	
Surgery time(min)	163.74 ± 47.23	124.97 ± 32.15	<0.001
Blood loss(ml)	680 (400)	424 (300)	<0.001
Liquid transfusion(ml)	2,269.81 ± 396.06	2,167.04 ± 510.76	0.179
Blood transfusion(yes/no)	23/30	50/129	0.033
Surgical procedures			
Anterior surgery	41 (77.36%)	138 (77.09%)	0.968
Posterior surgery	12 (22.64%)	41 (22.91%)	
Time from injury to surgery(days)	7.62 ± 2.93	5.51 ± 2.68	<0.001
D-dimer(mg/L)	2.60 (2.01)	1.88 (1.67)	0.011
FIB(g/L)	3.58 (1.29)	3.43 (1.23)	0.357
TT(s)	14.31 (2.05)	14.61 (2.20)	0.662
APTT(s)	29.49 (5.35)	29.17 (4.90)	0.537
PT(s)	12.25 (1.50)	11.93 (1.50)	0.226

BMI, body mass index; DVT, deep vein thrombosis; ASIA, American Spinal Injury Association; FIB, fibrinogen; PT, prothrombin time; TT, thrombin time; APTT, activated partial thromboplastin time.

### Multivariate analysis of DVT exacerbation or new DVT

Multivariate logistic regression was performed to identify risk factors. As shown in Table 3, the results showed that age (OR, 1.103; 95%CI:1.059–1.149; *P* < 0.001), ASIA score A-B (OR, 3.912; 95%CI:1.501–10.197; *P* = 0.005), time from injury to surgery (OR, 1.363; 95% CI:1.176–1.580; *P* < 0.001), surgery time (OR, 1.020; 95% CI:1.009–1.031; *P* < 0.001), and blood loss (OR, 1.001; 95% CI:1.000–1.003; *P* = 0.028) were risk factors for postoperative DVT exacerbation.

**Table 3:** Multivariable analysis of the risk factors for DVT exacerbation.

Risk factors	β	SE	Wald	Exp (β)	95% CI	*P* Value
Age(years)	0.098	0.021	22.616	1.103	1.059–1.149	<0.001
ASIA score	1.364	0.489	7.785	3.912	1.501–10.197	0.005
Surgery time(min)	0.020	0.006	12.550	1.020	1.009–1.031	<0.001
Time from injury to surgery(days)	0.310	0.075	16.973	1.363	1.176–1.580	<0.001
Blood loss(ml)	0.001	0.001	4.835	1.001	1.000–1.003	0.028

ASIA, American Spinal Injury Association.

### ROC analysis of different risk factors

**Figure 1:**
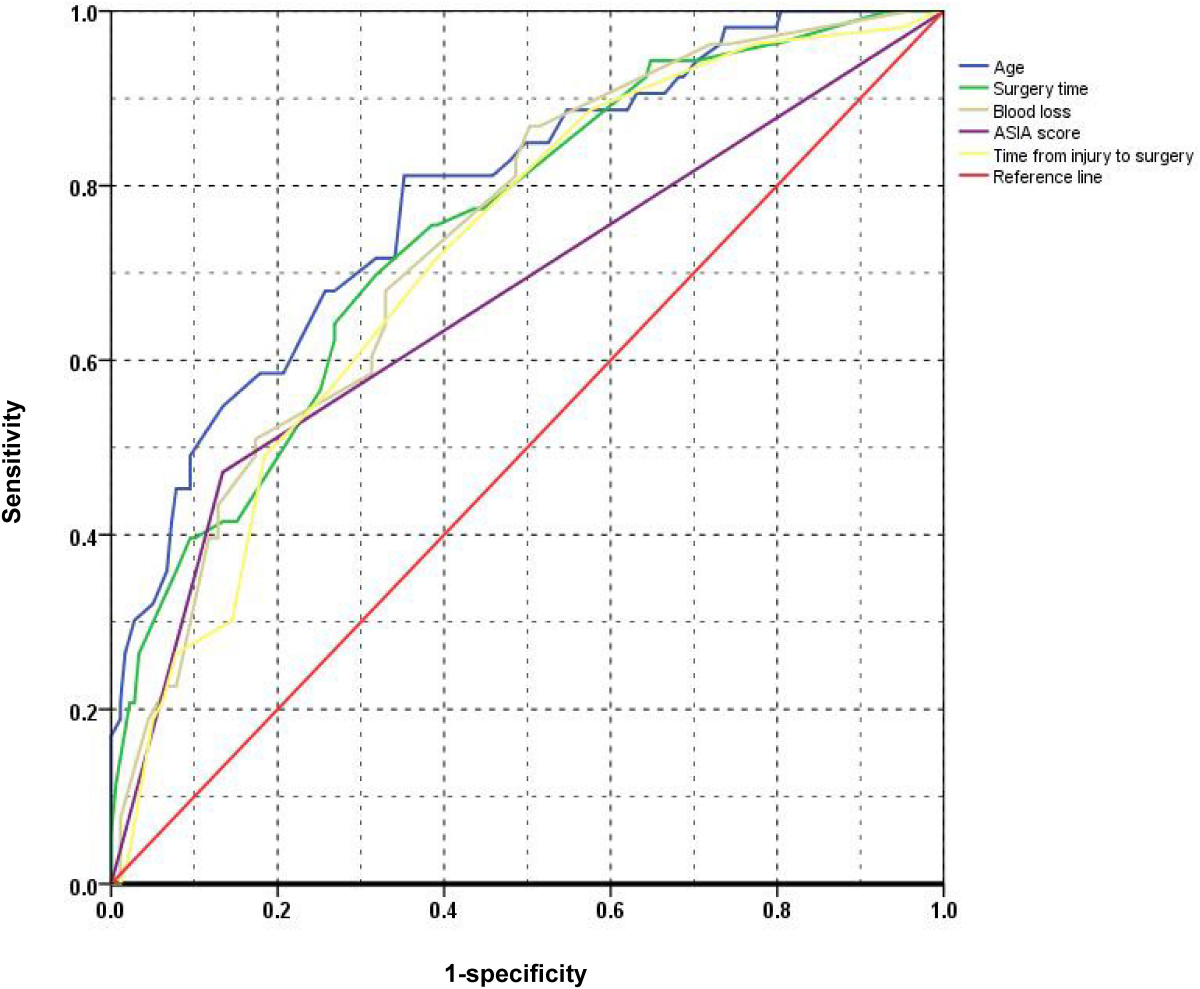
The ROC curves of different risk factors.

The ROC curves for the five risk factors were shown in Figure 1. The value of AUC (area under the ROC curve) usually represents the diagnostic value of risk factors. The diagnostic value of age (AUC value = 0.788) was the highest one among these factors. The AUC values of ASIA score, time from injury to surgery, surgery time, and blood loss were 0.669, 0.720, 0.750 and 0.740, respectively (Table 4). The diagnostic cut-off values were determined by the Youden index and were more than 5 days from injury to surgery, ASIA score A-B, 135 min of surgery time and 330 ml of blood loss, respectively.

**Table 4:** The ROC results of different factors.

Risk factors	Cut-off value^a^	Sensitivity	Specificity	AUC	95% CI	SE	*P* Value
Age(years)	50	81.13%	64.80%	0.788	0.730–0.839	0.036	<0.001
ASIA score	–	47.17%	86.59%	0.669	0.604–0.729	0.037	<0.001
Surgery time(min)	135	69.81%	68.16%	0.750	0.690–0.805	0.037	<0.001
Time from injury to surgery(days)	5	71.70%	60.89%	0.720	0.657–0.777	0.038	<0.001
Blood loss(ml)	330	86.79%	49.72%	0.740	0.678–0.795	0.037	<0.001

ASIA, American Spinal Injury Association.

^a^ The cut-off points of scores were determined by the Youden index.

## Discussion

Acute cervical SCI is often associated with cervical fracture, which can lead to serious complications (22). VTE is a common and serious complication after acute SCI. After SCI, dysfunction of the autonomic nervous system leads to lower limb venous vasodilation, increased venous blood flow resistance, decreased endothelial function, weakened platelet inhibition of thrombin production and prostaglandin release, enhanced coagulation function, and relatively weakened fibrinolysis function, resulting in blood being in a hypercoagulable state (10, 23–25). In addition, due to various reasons such as injury itself, limb paralysis after SCI, reduced lower limb activity, prolonged bed rest, surgery, and anesthesia, these are all conducive to the formation of DVT. Many studies have explored the incidence and risk factors of DVT after acute SCI, but mainly focused on the perioperative or postoperative period. They do not pay attention to DVT dynamic change after SCI, or do not comprehensively study the risk factors of DVT exacerbation. To our knowledge, this is the first retrospective study to evaluate the dynamic change of perioperative DVT in patients with SCI complicated with cervical fracture and analyze the risk factors of postoperative DVT exacerbation.

The acute occurrence of DVT is not static, but a dynamic process, which may involve the dissolution and aggravation of thrombus (26, 27). In our study, the majority of thrombi remain unchanged before and after surgery. But about 22.8% of patients experienced postoperative thrombus exacerbation, which was much higher than the patients with thoracolumbar fractures we previously studied (28). This situation may be related to severe muscle pump injury caused by spinal cord injury itself, blood hypercoagulability, and widespread muscle weakness.

In the current study, we found that age, ASIA score, time from injury to surgery, surgery time, and blood loss were risk factors for DVT exacerbation. Many studies found that the older the age, the higher the incidence of DVT in acute SCI patients (13, 15, 29). The results of a study demonstrated increased risk of DVT in acute traumatic SCI who were 50 years of age and older (30). Zhang et al. (15) showed that age had a linearly association with incidence of DVT for patients with SCI, and the incidence of DVT increased by 1.07-fold when age increased by 1 year. Our study found that patients older than 50 years were more likely to have DVT exacerbation after surgery. This is because with the increase of age, the patient's organs are in a state of decline, the blood flow is slow, the blood viscosity is increased, the concentration of clotting factors in the blood vessel endothelial cells is increased, and substances such as antithrombin are reduced, leading to the destruction of the coagulation-anticoagulation balance in the blood system, resulting in the formation of thrombosis (31).

Some studies have shown that loss of motor function is a risk factor for VTE in patients with cervical SCI (9, 10, 13, 22). We also found that ASIA score A-B was a risk factor for postoperative DVT exacerbation. Lower limb paralysis can weaken muscle pump function, reduce blood flow, and promote DVT. One report indicated that SCI above T6 can seriously affect the control of sympathetic nervous system over cardiac function, leading to a decrease in myocardial contractility, dilation of capillaries, gastrointestinal vascular beds, and coronary arteries, resulting in a reduction of approximately 50% in effective blood volume (10). This not only increases blood viscosity, but also greatly reduces muscle oxygen supply and muscle contraction, ultimately leading to a decrease in deep vein blood flow velocity in the lower limbs and an increase in the incidence of DVT (32). Our study found the time from injury to surgery greater than 5 days was closely associated with postoperative DVT exacerbation. Waiting for too long for surgery can prolong the patient's bed rest and braking time. After long-term bed rest, the venous blood flow of the lower extremities slows down or even stops, and the slow blood flow leads to the hypoxia of the valve, causing endothelial damage. Blood stasis can also cause the accumulation of local coagulation factors and the depletion of inhibitory factors, leading to the formation of DVT (10, 33).

We found that surgery time was a risk factor for postoperative DVT exacerbation. A meta-analysis showed that for patients undergoing spinal surgery, prolonging surgical time increased the incidence of DVT (34). This is because surgery can cause tissue factors to enter the blood circulation system, promote the activation of clotting factors, increase blood coagulation degree, but also cause platelet increase, adhesion and aggregation, and ultimately promote the formation and aggravation of DVT. The greater the amount of intraoperative blood loss, the lower the blood volume in the body, resulting in a decrease in blood flow velocity and blood stasis, which increases the risk of DVT. Our study found that increased intraoperative blood loss can lead to DVT exacerbation. We believed that blood loss disrupted the coagulation fibrinolysis system, and that fluid supplementation can modulate the coagulation system, which was consistent with Riha et al. (35). Therefore, by optimizing surgical procedures, improving the skills of surgeons and adequately supplementing fluids, the incidence of DVT exacerbation may be reduced.

The limitations of this study should be acknowledged. Firstly, this retrospective study has inherent limitations. Secondly, the small amount of data result in some variables that may be significantly related to deep vein thrombosis not being recorded or measured. Thirdly, D-dimer is not found to be associated with DVT exacerbation, as our study only included values at admission. Future prospective, large sample and multi-center studies are needed to confirm our results.

## Conclusions

In patients with spinal cord injury complicated with cervical fracture, the risk of postoperative DVT exacerbation is very high. Age, ASIA score A-B, time from injury to surgery, surgery time and blood loss were risk factors for postoperative DVT exacerbation or new DVT.

## Data Availability

The raw data supporting the conclusions of this article will be made available by the authors, without undue reservation.
